# Realistic anatomical model reconstruction and high-fidelity flow computation in stented coronary arteries for the analysis of in-stent thrombosis

**DOI:** 10.3389/fcvm.2026.1863952

**Published:** 2026-07-23

**Authors:** Ryo Torii, Amear Mohammed, Mazen Abou Gamrah, Ibrahim Halil Tanboga, Zaid A. Abdulelah, Anantharaman Ramasamy, Thamil Kumaran S. M., Tingquan Zhou, Xingwei He, Tom Crake, Janna Leung, Hui Ying Ang, Nicolas Foin, Jaryl Ng, Shawn Lian Shaoliang, Pieter Kitslaar, Jouke Dijkstra, James Moon, Andreas Baumbach, Christos V. Bourantas

**Affiliations:** 1 Department of Mechanical Engineering, University College London, London, United Kingdom; 2 Department of Cardiology, Barts Heart Centre, Barts Health NHS Trust, London, United Kingdom; 3 Atlas University Medical School, Department of Cardiology and Biostatistics, Istanbul, Turkey; 4 William Harvey Research Institute, Queen Mary University London, London, United Kingdom; 5 Medical School, University of Birmingham, Birmingham, United Kingdom; 6 National Heart Centre Singapore, Singapore, Singapore; 7 Duke-NUS Medical School, Singapore, Singapore; 8 Medis Medical Imaging Systems, Leiden, Netherlands; 9 Division of Image Processing, Department of Radiology, Leiden University Medical Centre, Leiden, Netherlands; 10 Institute of Cardiovascular Sciences, University College London, London, United Kingdom; 11 Homerton University Hospital, Homerton Healthcare NHS Foundation Trust, United Kingdom

**Keywords:** coronary artery anatomical model reconstruction, endothelial shear stress, optical coherence tomogaphy, shear rate, stent thrombosis

## Abstract

**Background and aims:**

This article aims to develop and validate a methodology for realistic anatomical reconstruction of stented segments from optical coherence tomography (OCT) and angiographic images and the computation of local haemodynamic forces using computational fluid dynamics, towards a more realistic haemodynamic characterisation in coronary atherosclerotic lesions post-PCI.

**Methods:**

Three-dimensional anatomical models of stented vessels (6 silicone models *in vitro* and 16 patient models) were reconstructed from OCT and angiography, using a new methodology called REFINED, which relies on the separate reconstruction of the lumen and the stent; these two are then fused in a single model. Blood flow simulations were performed using the anatomical models to calculate the endothelial shear stress (ESS) and shear rate (SR). The models were geometrically and haemodynamically evaluated against those reconstructed using conventional models.

**Results:**

The reconstruction error distance was smaller with the REFINED approach than with the conventional approach, in both the silicone and patient models [8 (2–15) μm vs 23 (10–54) μm, *p* < 0.001, and 19 (9–32) μm vs 19 (7–36) μm, *p* < 0.001]. The REFINED method depicted higher ESS at the top of the struts in both the silicone (8.01 (4.21–14.92) Pa vs 5.54 (2.96–9.63) Pa; *p* < 0.001) and patient models [2.41 (1.41–3.65) Pa vs 1.44 (0.77–2.52) Pa; *p* < 0.001], and a more spatially heterogeneous SR compared to the conventional approach (*p* < 0.001 for all analyses).

**Conclusions:**

The REFINED approach outperforms the conventional method in reconstructing stent geometry and has been shown to be effective in facilitating a high-fidelity computation of the highly disturbed local haemodynamic environment. This demonstrates its potential role in examining device failure.

## Introduction

Local haemodynamic forces, in particular endothelial shear stress (ESS) and shear rate (SR), are well-known modulators of the vessel wall healing response following percutaneous coronary interventions (PCI) and have been considered instigators of restenosis, neo-atherosclerosis and stent thrombosis (1–6). Several clinical studies have shown that low ESS post-stent implantation is associated with neointimal growth and the formation of lipid-rich neoatherosclerotic lesions, while high ESS in stented segments has been identified as a predictor of their destabilisation and rupture (5, 7–9). In addition, experimental studies have suggested that pathological low or high SR can promote thrombus formation and stent occlusion (1, 4, 6).

The *in vivo* assessment of flow patterns has become feasible with the advent of intravascular imaging techniques. The first studies fused intravascular ultrasound (IVUS) and angiographic data to reconstruct stented segments, which were then processed with computational fluid dynamics (CFD) techniques to derive flow patterns (7, 8, 10). Nevertheless, the image resolution of these models was too limited to allow precise modelling of stented segment geometry. In contrast, optical coherence tomography (OCT), with its unparalleled resolution, enables the detailed depiction of struts and the evaluation of their impact on ESS distribution (3, 11). However, the first methodologies that used OCT imaging to define lumen geometry post-stenting had significant limitations, as they generated a single surface of the stented segments. This was based on the assumption that the stent struts were well apposed, which resulted in the smoothening of the stent edge (3, 12). To address these limitations, several methodologies have since been introduced that can produce an accurate representation of stented segments by separately reconstructing the stent and lumen geometries and fusing them afterwards (13–21). However, a comprehensive assessment of their efficacy and accuracy against the conventional reconstruction method is still lacking in terms of both reproducing stented coronary anatomy and its impact on the local haemodynamics in a clinical setting (14, 19).

The aims of the present study are to describe a new methodology for reconstructing stented segments based on the separate modelling and fusion of vessel and stent geometry, and to validate its accuracy *in vitro* and *in vivo* by comparing the distribution of the local haemodynamic forces in models reconstructed using the new method and a well-established approach that generates a single lumen and stent surface geometry.

## Materials and methods

### *In vitro* study

We used the data from a previously published study (1) for our *in vitro* validation. Here, six stenotic vessel models (each with a reference diameter of 2.75 mm and 40% stenosis in their middle) made from silicone elastomer were stented with a 3.0 × 18 mm sirolimus-eluting stent (Orsiro, Biotronik, Germany) and perfused with porcine blood for 60 min at a flow rate of 200 mL/min, after which OCT imaging was performed to image the vessel model, stent, and thrombosis generated in the models. In three of the six models, the stents were deliberately under-expanded to generate stent malapposition. Further details of the *in vitro* study can be found in the Supplementary Material and in the cited study by Ng et al.

### *In vivo* study

We retrospectively analysed data acquired from 16 patients who underwent PCI with an Xience Alpine (Abbott Vascular Inc, Santa Clara, CA, USA) or Synergy™ (Boston Scientific, Natick, Massachusetts, USA) everolimus-eluting stent and OCT following stent implantation. OCT was performed with a C7XR or an OPTIS™ system (Abbott Vascular Inc, Santa Clara, CA, USA) using an automated pull-back device at a constant speed (20 mm/s for the C7XR and 18 mm/s for the OPTIS) during continuous injection of contrast medium (frame rates: 100 fr/s for the C7XR and 180fr/s for the OPTIS system). Patients undergoing PCI in the left main stem or in a bifurcation lesion using the culotte or double kiss-and-crush technique and those with suboptimal angiographic projections for coronary artery reconstruction (e.g., due to foreshortening or overlapping) or a TIMI frame count of ≤2 post-PCI were excluded from the analysis. The study was reviewed and approved by the local research ethics committee (REC reference: 23/HRA/4714).

### OCT data processing

**Figure 1:**
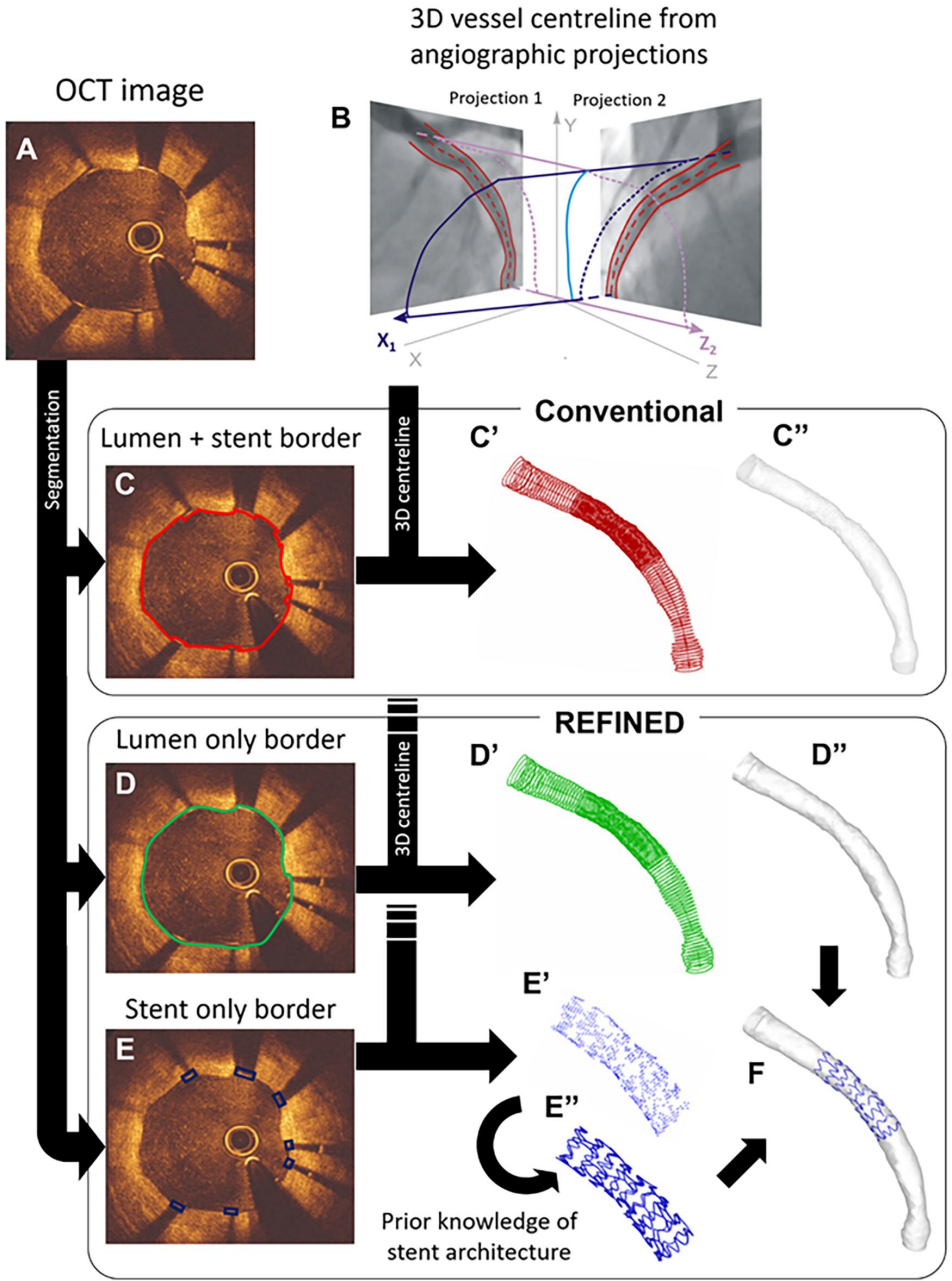
Reconstruction of a silicone model with a thrombus proximal to the stenosis site, using the conventional and the REFINED approaches **(A,B)**. The location of the thrombus volume is indicated by red shading in panels C and D, while panels E, F, G and H show the streamlines and SR distribution in the two reconstruction models. It is apparent that, at the thrombus site, there are malapposed struts that lead to high and heterogeneous SR regions, which are depicted only by the REFINED method. Panel I shows the SR distribution at the thrombus and non-thrombus sites across the entire set, Panel J shows the percentage of the volume exposed to different levels of SR (<300 s^−1^, >1000 s^−1^ and >2000 s^−1^) in the two cases where the thrombus was present at the location of the stenosis, and in the four cases where the thrombus was present proximally to the stenosis, across the two reconstruction approaches.

The OCT data collected *in vitro* and *in vivo* were analysed offline using proprietary software (QCM-CMS Medis Medical Imaging Systems, Leiden, The Netherlands) following the procedure previously established (2). Details of the method can be found in the Supplementary Material; however, briefly, separate identification and delineation of (i) the unified lumen and stent border, (ii) the lumen-only border, (iii) the strut centre point and (iv) the thrombus volume (only for *in vitro* models) were conducted per OCT frame, as shown in Figure 1. All OCT frames were used for the *in vitro* model, and for the *in vivo* models, OCT frames within the stented segment at 0.2 mm intervals and those within the non-stented segment at 0.4 mm intervals were used.

### Reconstruction of an anatomical model

Three-dimensional coronary anatomical model reconstruction was performed using two methodologies (Figure 1). The first one, the conventional approach, utilised the lumen areas for the non-stented segment and the lumen-stent border for the stented segment to define the lumen geometry. The reconstruction steps were previously established (3) and are briefly described below (the full process is explained in The Supplementary Material). The 3D vessel centreline was extracted from two end-diastolic angiographic projections, using 3D quantitative coronary angiography (3D-QCA) software QAngioXA3DRE (Medis Medical Imaging Systems, Leiden, the Netherlands). For silicone models, no angiographic images were acquired, and the centreline was approximated by a straight line, as the models were straight tubes. The lumen and the unified lumen-stent borders were then placed on the 3D vessel centreline, with frame twist corrected based on anatomical landmarks, e.g., side branches, to which a 3D surface was fitted to represent the 3D stented vessel. In the presence of side branches with a diameter > 1 mm, 3D-QCA was performed to generate the side branch geometry and the reconstructed 3D models were fused.

The second methodology, named REFINED (Reconstruction of stEnt vessel model by Fusion of INtravascular imagEs and caD), involves the separate reconstruction of the lumen and stent geometry. The reconstruction of the lumen was performed following the same steps described above, using, on this occasion, the lumen-only borders and not the unified lumen-stent borders in the stented segment (Figure 1D). Then, the 3D coordinates of the annotated stent struts were extracted using the same reconstruction methodology, and the 3D points corresponding to the strut locations in space were manually connected with 3D spline curves to generate the stent skeleton, using prior knowledge of the stent's architecture. This information, along with the stent dimensions, was then used to create the stent CAD model, incorporating the known strut cross-sectional profile and thickness. This model is then fused with the 3D lumen model to construct the final stented vessel model (Figure 1).

### Blood flow simulation and data analysis

The reconstructed anatomical models were processed further to be used in blood flow computations. Proprietary software (ANSYS ICEM CFD and CFX version 2019 R3, Ansys Inc., Canonsburg, PA, USA) was used for meshing and flow simulations, respectively. A mesh independence test was conducted as per the expert recommendation (4). To capture the detailed characteristics of the flow patterns, the mesh density was increased near the stent struts, with a maximum element edge equal to approximately 1/4 of the strut thickness (i.e., ∼20 μm).

In accordance with the experimental conditions, a steady flow of 200 mL/min was applied to the proximal end of the silicone models (1). For the *in vivo* models, a typical pulsatile velocity waveform, as measured using an intravascular Doppler wire, was extracted from the literature (5), then scaled to have a patient-specific mean flow speed and applied at the proximal end as the inflow condition. Here, the patient-specific flow speed was estimated by measuring the number of frames required for the contrast dye to pass from the inlet to the outlet of the reconstructed segment, the length of the segment and the cine frame rate in two angiographic projections (6).

Blood was modelled as a non-Newtonian fluid by applying the Quemada equation to account for its shear-thinning rheological characteristics (7). The haematocrit was set based on the literature, at 41% (8) for the *in vitro* models (swine blood) and at 43% (9) for the *in vivo* models. Blood density was assumed to be 1,060 kg·m^−3^ for all cases. The vessel and stent walls were considered rigid, and no-slip conditions were imposed. The flow rate split between the main and side branch outlets was estimated using a previously described scaling approach based on the diameters of the main vessel and side branches (10, 11).

### Geometrical and haemodynamic evaluation of the two approaches in reconstructing stented segment geometry

The performance of the two approaches to reconstruct stented segments was assessed *in vitro* and *in vivo* using the error distance, which is defined as the difference between the 3D coordinates of the original stent struts (= reference point) and the nearest point of the reconstructed model. This also depends on the apposition status of the stent strut. Further details can be found in the Supplementary Material.

**Figure 2:**
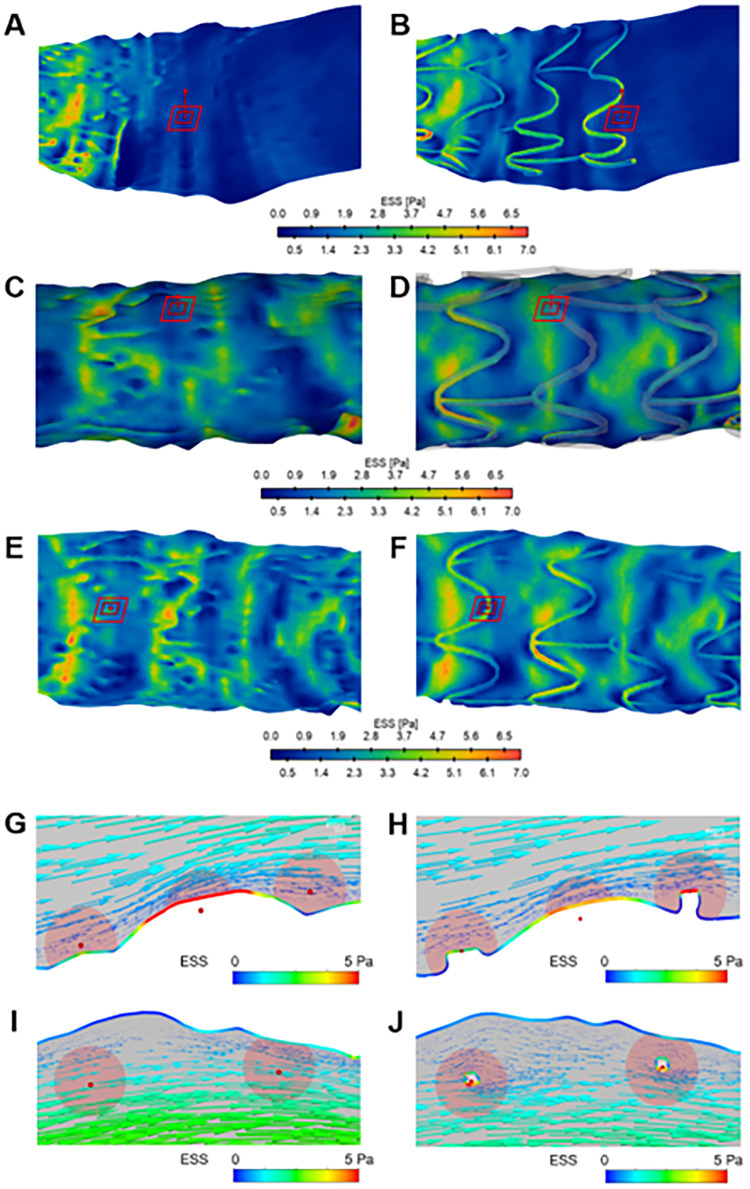
3D model of a case with a bifurcation lesion treated using the TAP technique, reconstructed with the conventional **(A)** and REFINED **(B)** methods. The insert panels show the region of high shear rate (>1000 s^−1^) at the time of peak flow.

The impact of the two reconstruction approaches on flow patterns was evaluated by measuring the ESS (i) on the struts (sampling area = strut thickness) and (ii) around the struts (sampling area = 400 μm), as illustrated in Figure 2. The SR values were computed around the struts in spheres with a diameter of 320 μm and centred at the centre of each strut. The sampling area and volume for the ESS and SR computation were selected after performing a sensitivity analysis of two randomly selected stent models. ESS and SR values were assessed in different areas/volumes, and the results showing the largest difference between the two approaches were reported to underscore the limitations of the conventional approach to assessing flow patterns against the REFINED methodology. Where geometrical correspondence could not be found, such as in the case of a malapposed strut reconstructed by the REFINED approach, the variables at the closest point of the model surface were sampled for comparison. A detailed definition of these sampling areas can be found in the Supplementary Material. In addition to the ESS (and time-averaged ESS for *in vivo* models) and SR, other commonly used multidirectional ESS variables were considered, including oscillatory shear index (OSI), relative residence time (RRT), transverse ESS (transESS) and cross-flow index (CFI) (12) for *in vivo* models. These are presented in the Supplementary Material.

### Statistical analysis

Continuous variables are presented as medians and quartiles, while categorical variables are presented as absolute values and percentage of the total sample size. Error distances of the two reconstruction models by the two methods – conventional and REFINED – in reference to the original (OCT-image-based) strut point were compared using a Wilcoxon signed-rank test; the same approach was used to compare the ESS and SR estimations from the two approaches. The median difference between the estimations of the two reconstruction methods for the above metrics was computed using Hodges–Lehmann estimators and their 95% confidence intervals. All statistical tests were two-tailed, and statistical significance was set at 0.05. The analyses were performed using the R software version 4.2.2 (R statistical software, Institute for Statistics and Mathematics, Vienna, Austria).

## Results

### Patients studied

The baseline characteristics of the patients included in this study are shown in Supplementary Table 1. The majority of the patients were men who suffered from hypertension and hypercholesterolaemia and had a preserved left ventricular systolic function. All underwent PCI for *de novo* disease; 14 had treatment with a single stent, while two underwent PCI for a bifurcation lesion with a 2-stent strategy (T- and small-protrusion techniques).

### Efficacy of the 3D reconstruction approaches to model stent geometry

Successful reconstruction of all silicone models produced 8,970 strut points in total, of which 7,097 were classified as apposed, 20 as embedded, and 1,853 as malapposed. Error distances, presented in Table 1, were significantly larger for the conventional approach than those in the REFINED approach, irrespective of the apposition status (*p* < 0.001 for all apposition statuses; see also Supplementary Figure 2).

**Table 1:** Error distances between the original strut points in OCT, transferred in 3D space, and the reconstructed stent models using the two approaches, in the silicone models and the human data. The H-L estimates represent the median difference between the conventional and REFINED approaches.

	Conventional	REFINED	H-L estimate (95% CI)	p-value
***In vitro* data**				
Entire set of struts (*μ*m)	23 (10, 54)	8 (2, 15)	20 (19, 21)	<0.001
Apposed struts (μm)	18 (9, 34)	8 (2, 15)	12 (11, 13)	<0.001
Embedded struts (μm)	23 (10, 34)	7 (2, 12)	17 (9, 26)	<0.001
Malapposed struts (μm)	136 (42, 226)	8 (2,15)	131 (−126, 137)	<0.001
**Human data**				
Entire set of struts (μm)	19 (7, 36)	19 (9, 32)	2 (1, 2)	<0.001
Apposed struts (μm)	16 (6, 30)	19 (9, 32)	−2 (−3, −2)	<0.001
Embedded struts (μm)	29 (15, 49)	19 (10, 32)	10 (9, 11)	<0.001
Malapposed struts (μm)	138 (43, 253)	24 (12, 36)	123 (113, 134)	<0.001

Reconstruction of human models was also successfully performed in all cases, producing 17,917 strut points in total, of which 13,164 were classified as apposed, 3,654 as embedded, and 1,099 as malapposed. Error distances (Table 1) were statistically smaller in the conventional approach in apposed struts compared to those in the REFINED approach, but these differences were marginal (median 16 μm vs 19 μm, *p* < 0.001). Conversely, in embedded and malapposed struts, the error distances were significantly smaller in the models reconstructed using the REFINED approach (*p* < 0.001 for both, Table 1 and Supplementary Figure 2). It should be noted that the embedded and malapposed struts are not present in the 3D models reconstructed using the conventional approach. In these cases, the error distance corresponds to the distance from the original OCT-based strut point in 3D space to the nearest lumen border. An example of the reconstructed models is also shown in Supplementary Movie 1 as a fly-through animation.

### Impact of the reconstruction approach on the ESS estimations

#### *In vitro* analysis

The ESS distributions in the silicone models are shown in Table 2. In apposed struts, ESS values were found to be smaller in the conventional approach compared to the REFINED method at the top of the struts, but higher in the areas around the struts (*p* < 0.001 for both). Conversely, there were no significant differences in the ESS metrics for embedded struts (*p* = 0.538 and *p* = 0.723, respectively), whereas in malapposed struts, the ESS were lower in the REFINED approach (*p* < 0.001) and higher in the area around the projected struts (*p* < 0.001). The SR values were higher in the conventional approach in apposed struts compared to the REFINED approach, but lower in the embedded and malapposed struts. Irrespective of the strut apposition status, the standard deviation of the SR values in the 320 μm sampling spheres was significantly higher in the REFINED method than in the conventional approach.

**Table 2:** ESS and SR estimations based on the two reconstruction methods.

	Conventional	REFINED	H-L estimate (95% CI)	p-value
*IN VITRO*				
**ESS at the top of the struts**				
Entire set of struts (Pa)	5.54 (2.96, 9.63)	8.01 (4.21, 14.92)	−2.65 (−2.77, −2.53)	<0.001
Apposed struts (Pa)	5.53 (2.96, 9.89)	9.14 (5.57, 16.82)	−3.64 (−3.78, −3.51)	<0.001
Embedded struts (Pa)	4.93 (2.82, 9.89)	6.29 (1.75, 8.38)	0.62 (−1.28, 2.92)	0.538
Malapposed struts (Pa)	5.55 (2.96, 8.57)	3.54 (1.07, 7.98)	1.34 (1.11, 1.58)	<0.001
**ESS around the struts**				
Entire set of struts (Pa)	4.79 (2.76, 8.45)	4.24 (2.40, 8.19)	0.57 (0.52, 0.62)	<0.001
Apposed struts (Pa)	4.52 (2.68, 8.48)	3.81 (2.31, 7.36)	0.79 (0.73, 0.84)	<0.001
Embedded struts (Pa)	7.60 (3.27, 11.78)	6.22 (3.52, 9.94)	0.40 (−0.73, 2.14)	0.723
Malapposed struts (Pa)	5.57 (3.35, 8.24)	6.87 (3.47, 10.44)	−0.69 (−0.88, −0.50)	<0.001
**SR**				
Entire set of struts (s^−1^)	1348 (940, 2004)	1263 (755, 2199)	68.7 (57.8, 79.4)	<0.001
Apposed struts (s^−1^)	1350 (909, 2068)	1136 (727, 2030)	126.8 (116.6, 137.0)	<0.001
Embedded struts (s^−1^)	2414 (1464, 3097)	1812 (1315, 2570)	235.9 (−89.6, 703.2)	0.198
Malapposed struts (s^−1^)	1342 (1116, 1731)	1857 (1028, 2770)	−385.2 (−442.0, −330.4)	<0.001
**Standard SR deviation**				
Entire set of struts (s^−1^)	255 (159, 463.5)	551 (327, 976)	−256.1 (−264.2, −248.0)	<0.001
Apposed struts (s^−1^)	255 (159, 457)	479 (306, 768)	−192.2 (−198.8, −185.7)	<0.001
Embedded struts (s^−1^)	904 (584, 1130)	743 (645, 877)	95.2 (−60.3, 253.2)	0.225
Malapposed struts (s^−1^)	256 (159, 480)	1157 (642, 1568)	−733.1 (−769.1, −697.7)	<0.001
***IN VIVO***				
**TAESS at the top of the struts**				
Entire set of struts (Pa)	1.16 (0.61, 2.01)	1.84 (1.07, 3.03)	−0.77 (−0.79, −0.75)	<0.001
Apposed struts (Pa)	1.21 (0.62, 2.14)	2.19 (1.36, 3.39)	−0.86 (−0.88, −0.83)	<0.001
Embedded struts (Pa)	1.10 (0.67, 1.66)	1.15 (0.74, 1.73)	−0.14 (−0.16, −0.12)	<0.001
Malapposed struts (Pa)	0.78 (0.37, 1.70)	0.72 (0.31, 1.85)	−0.03 (−0.19, 0.12)	0.680
**TAESS around the struts**				
Entire set of struts (Pa)	1.18 (0.74, 1.77)	1.19 (0.76, 1.78)	−0.002 (−0.003, −0.002)	<0.001
Apposed struts (Pa)	1.17 (0.73, 1.76)	1.17 (0.74, 1.75)	−0.002(−0.003, −4 × 10^−4^)	0.035
Embedded struts (Pa)	1.24 (0.82, 1.75)	1.27 (0.89, 1.80)	−0.001(−0.001, −0.001)	<0.001
Malapposed struts (Pa)	0.99 (0.61, 1.94)	1.02 (0.58, 2.06)	−4 × 10^−4^(−5 × 10^−4^, −3 × 10^−4^)	<0.001
**SR**				
Entire set of struts (s^−1^)	345.5 (225.3, 499.5)	341.9 (221.0, 497.5)	10.9 (10.0, 11.8)	<0.001
Apposed struts (s^−1^)	353.5 (226.5, 514.0)	334.4 (211.4, 489.0)	21.6 (20.7, 22.6)	<0.001
Embedded struts (s^−1^)	338.6 (237.0, 481.5)	368.2 (264.7, 503.9)	−20.9 (−22.7, −19.1)	<0.001
Malapposed struts (s^−1^)	270.4 (181.0, 397.1)	337.4 (196.5, 621.8)	−60.5 (−78.7, −45.5)	<0.001
**Standard SR deviation**				
Entire set of struts (s^−1^)	69.8 (41.9, 120.0)	115.0 (66.5, 191.2)	−40.1 (−41.1, −39.2)	<0.001
Apposed struts (s^−1^)	70.7 (42.4, 117.6)	119.8 (70.4, 192.7)	−45.5 (−46.5, −44.5)	<0.001
Embedded struts(s^−1^)	70.7 (43.6, 134.1)	80.4 (50.6, 140.9)	−4.88 (−5.92, −3.85)	<0.001
Malapposed struts (s^−1^)	53.6 (30.8, 103.0)	211.5 (127.8, 386.6)	−161 (−172, −151)	<0.001

#### *In vivo* analysis

The findings from the CFD analyses performed on 16 patients following stent implantation are also summarised in Table 2, and some typical examples are shown in Figure 2, with indication of the sampling area/volume of biomechanical metrics, such as TAESS. Illustrative examples of biomechanical metrics on the luminal surface are also shown in Supplementary Figure 3 in the form of a spread-out plot, with a 3D version of the comparison of TAESS shown in Supplementary Movie 2. Additionally, an animation showing pulsatile flow is shown in Supplementary Movie 3.

In models reconstructed using the conventional approach, TAESS values at the top of the struts were lower in apposed struts compared to the REFINED approach (*p* < 0.001); conversely, in embedded and malapposed struts, TAESS estimations at the top of the struts were numerically similar in the two reconstruction methods. Similar TAESS values were also reported in models reconstructed using either approach in areas around the struts, irrespective of strut apposition. In these models, even the ranges of the biomechanical variables provided by the two methods are close, e.g. TAESS around apposed struts, the difference reaches statistical significance due to the large sample size (e.g. 13,164 apposed strut points).

Significant differences were noted in the multidirectional ESS metrics (Supplementary Table 2). In models reconstructed with the conventional approach, the RRT was higher at the top of apposed struts, numerically similar in embedded struts, and lower in malapposed struts at the lumen surface at the location of the projected strut compared to the REFINED approach. Conversely, in the areas around the struts, the RRT values were higher in the REFINED approach in apposed and malapposed struts, but these are numerically similar to the estimations of the conventional approach for the embedded struts. The spatial distribution of the RRT (Supplementary Figure 3) indicated that there are regions of high RRT behind the strut, captured clearly by the REFINED approach, but not by the conventional method. The OSI values were close to 0 in all the apposition categories in both methodologies. In the conventional approach, the TransESS values were lower at the top of apposed struts but numerically similar to those in embedded and malapposed struts at the lumen surface at the location of the projected struts. Similar TransESS values were noted between the estimations of the two methods in the areas around the struts, irrespective of the strut apposition status. Finally, the CFI values were numerically similar in the two reconstruction methods in all the apposition categories.

**Figure 3:**
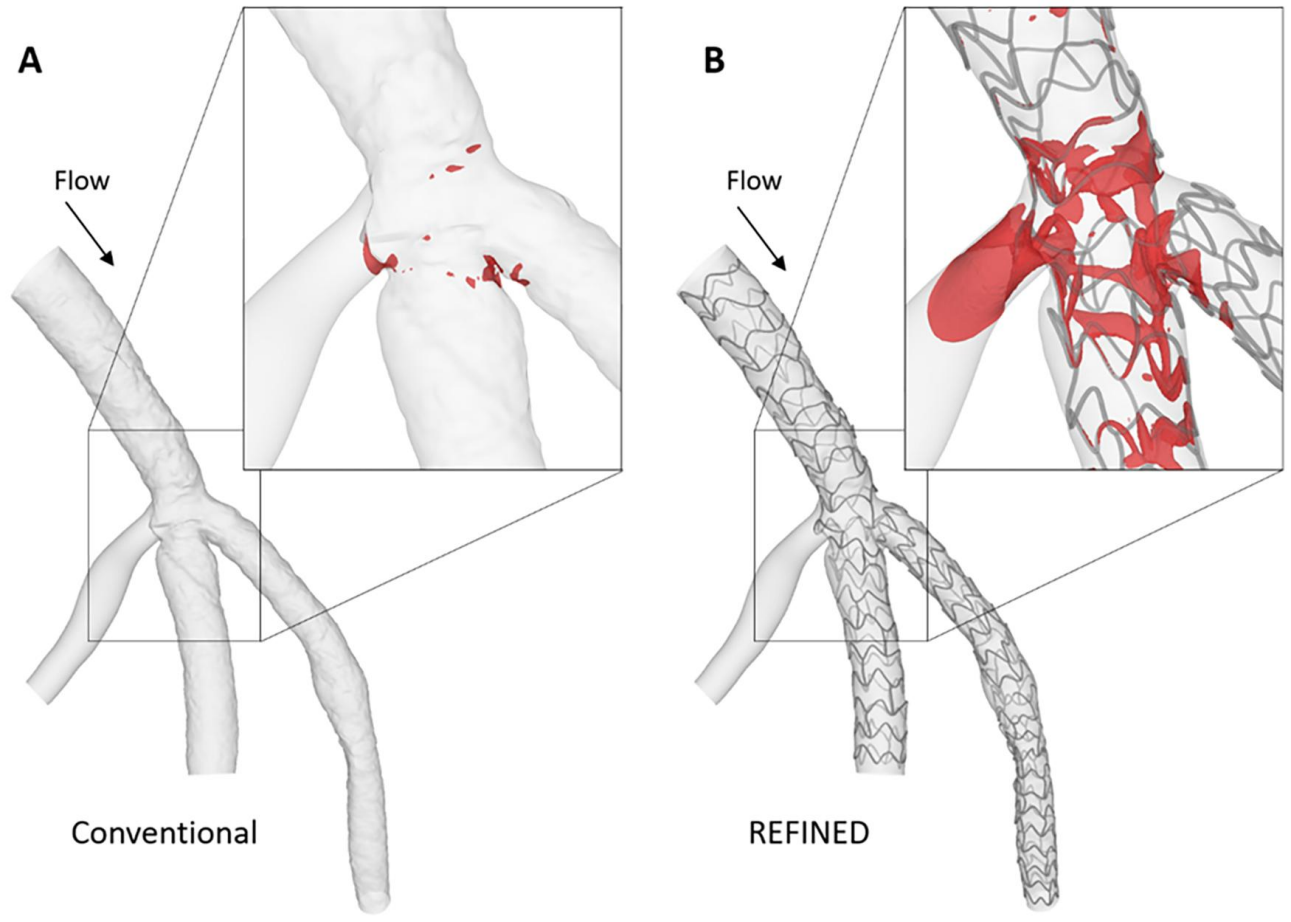
Methodology implemented to measure the ESS in the models reconstructed by the conventional and the REFINED approaches. Irrespective of the strut apposition status in the conventional approach, the 3D footprint of the struts was used to identify the closest point of the lumen-stent model, and this was used to define two squares: the first with an edge equal to the strut thickness and the second with an edge of 400 μm **(A,C,E)**. The same approach was also followed in the REFINED approach for the malapposed and embedded struts **(B,D)**, while in the case of apposed struts, the two squares were drawn around the stent point that was closer to the 3D footprint of the struts **(F)** The ESS and multidirectional ESS were then measured in the small square and in the area defined between the large and small squares. In both approaches, the SR was measured in a sphere with a diameter of 320 μm centred on the 3D location of the strut, irrespective of the strut apposition status **(G,H,I,J)**.

The SR values were higher in the conventional approach in apposed struts compared to the REFINED approach and lower in embedded and malapposed struts. In apposed and malapposed struts, the standard deviation of the SR in the 320 μm sphere was significantly higher in the REFINED method than the conventional approach, while the standard deviation was found to be numerically similar for embedded struts (Table 2). It should be noted that, even for an embedded strut, part of the 320 μm sampling sphere included some flow volume, as shown in Figures 2G,H; hence, the SR values are non-zero. An example of SR distribution in a bifurcation lesion is shown in Figure 3.

### Haemodynamic variables in thrombosed and non-thrombosed segments *in vitro*

**Figure 4:**
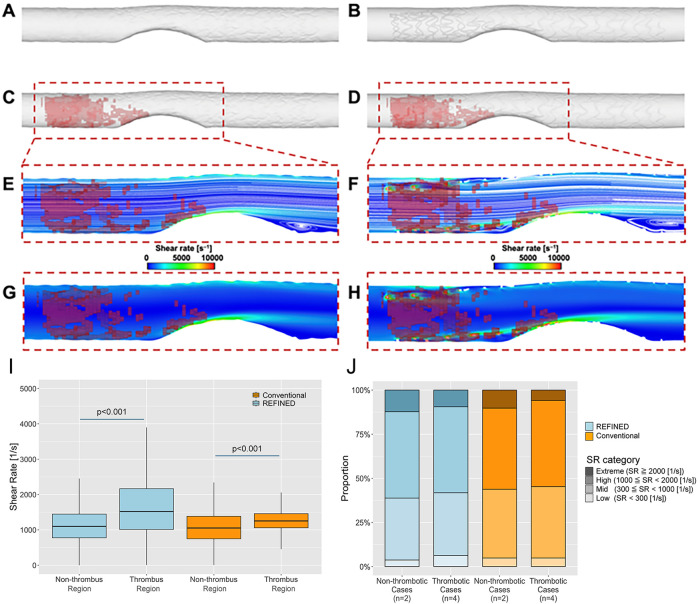
Conventional and REFINED methodologies for coronary artery reconstruction. In the conventional approach, the lumen borders in the non-stented segment and the stent borders in the stented segment are annotated in OCT images **(A,C)**, and then placed perpendicular to the lumen centreline, which is extracted from two angiographic projections **(B)**, to generate a point cloud (C′) that provides the basis for the generation of the 3D lumen-stent geometry surface (C′′). The REFINED approach involves the annotation of the lumen borders in the native and stented segments **(D)** and the identification of the struts in the stented segment **(E)** The lumen and stent borders are placed on the lumen centreline extracted from two angiographic projections and the former are used to generate the basis for the lumen silhouette (D′) first, and ultimately the lumen surface (D′′) while, the latter leads to the stent skeleton (E′), which provides the basis for the construction of the stent geometry (E′′). The lumen and the stent models are then combined to generate the final lumen-stent reconstruction **(F)**.

A thrombus was identified at the stenosis site in two models and proximally to the stenosis at the location of malapposed struts in four models (an example shown in Figure 4). The mean thrombus volume at the site of the stenosis was 1.21 mm^3^ and 8.70 mm^3^ proximal to the stenosis. The median SR at the thrombus site was 1257 (445, 2089) s^−1^ in the conventional approach and 1564 (8.5, 4195) s^−1^ in the REFINED approach (*p* < 0.001) and was higher than in the non-thrombus segment [1054 (7.3, 2347) s^−1^ and 1101 (1.6, 2461) s^−1^, *p* < 0.001, Figure 3].

Figure 4 also shows the ratio of regions with high SR in the models. In the two models where thrombus was identified in the stenosis, more extensive high SR regions were captured by the REFINED approach (61% of the total region was >1000 s^−1%^ and 12% was >2000 s^−1^) than the conventional approach (56% and 10%, respectively). Meanwhile, 3.7% and 4.8% of the total region were shown as exposed to low shear rate (<300 s^−1^) in the REFINED and conventional approaches, respectively. In the four models where thrombus was formed proximally to the stenosis and malapposition was noted, more extensive high SR regions were captured by the REFINED approach (58% of the total region was >1000 s^−1^ and 9.5% was >2000 s^−1^) than the conventional approach (55% and 5.8%, respectively). For low shear regions, the REFINED approach showed a larger extent, 6.2% of the total region, than the conventional (4.8%), indicating higher SR heterogeneity in the models reconstructed by the REFINED method.

## Discussion

We presented the REFINED methodology for the realistic reconstruction of stented segment geometry from x-ray angiography and OCT based on the separate reconstruction of lumen and stent models. The reconstructed model and flow patterns simulated in the models were compared against a conventional approach, which models one surface combining lumen and stent geometry. *In vitro* and *in vivo* validations demonstrated that the REFINED method enables the accurate reconstruction of stent architecture and outperforms the conventional approach, especially in the reconstruction of malapposed struts. This markedly affected the distribution of haemodynamic variables, with the REFINED method showing a higher ESS at the top of the struts and a more spatially heterogeneous SR around the struts. Differences in SR metrics between the two methods were noted in the silicone models when the analysis focused on thrombosed and non-thrombosed segments. This finding underscores the limitations of the conventional approach in accurately assessing flow disturbances, potentially impacting the prediction of clinical events.

Local haemodynamic forces are known to play a key role in the response of the vessel wall following stent implantation. Several CFD studies in models reconstructed from intravascular imaging data have shown that low ESS instigates neointimal proliferation and the formation of neoatherosclerotic plaques, whereas high ESS contributes to their destabilisation (6, 13–15). In addition, cumulative data have indicated that flow disturbances and high SR can cause the unfolding of the von Willebrand factor and platelet activation, which may lead to stent thrombosis (16–19). The medical device industry has attempted to optimise in-stent haemodynamic profiles using both benchtop experiments and CFD. These studies have demonstrated that thinner struts with a circular shape, large inter-strut spaces, and strut connectors parallel to the blood flow are associated with an optimal haemodynamic milieu (20–23). More recently, the effects of scaffold design and strut embedment state on *in vivo* ESS distribution have also been shown for different bioresorbable scaffolds (24–26). However, these studies had significant methodological limitations. Vessel models were reconstructed using a conventional method that generates a unified surface model of the lumen and stent. This method was unable to capture the impact of detailed scaffold design on the local haemodynamic milieu, especially the effects of struts that are malapposed or struts over the side branch orifice. These limitations become more evident when comparing flow patterns after the implantation of stents that have thinner struts. A recent report comparing flow patterns following ultrathin strut and thin strut bioresorbable polymer drug-eluting stent implantation failed to demonstrate an effect of the different strut thickness on ESS distribution (27).

In recent years, several studies have proposed the separate modelling of the lumen and stent geometry and the fusion of these two surfaces in a single model (2, 28–35). However, some of these reports included a single case (28, 35) others were applied only *in vitro* (30–32), and others lacked a detailed validation using human data (33, 36). Moreover, reports that included *in vivo* validation either did not examine ESS and SR patterns in relation to stent strut apposition (29) or did not incorporate the side branches into the 3D vessel geometry and did not examine the implications of strut protrusion on multidirectional ESS (2, 29).

This study overcomes the above limitations as it introduced a user-friendly framework that enables the fast and realistic reconstruction of stented segment geometry. Its accuracy was assessed *in vitro* and *in vivo* against a conventional reconstruction approach using a thorough validation methodology. We found that the REFINED approach facilitated more accurate reconstruction of stented segments than the conventional approach, as it was capable of reconstructing malapposed and embedded struts with high accuracy, with a mean error distance of 8 (2–15) µm *in vitro* and 19 (9–32) µm *in vivo*. The larger error *in vivo* was likely due to motion artefacts that are often seen in OCT images, caused by the longitudinal and lateral motion of the catheter relative to the vessel wall (37), which resulted in frame-to-frame inconsistency of OCT images and distortion of the reconstructed stent geometry. Despite these challenges, the distance discrepancy was within the scale of the OCT image resolution, indicating that the REFINED method enables accurate modelling of stent architecture.

The difference in model reconstruction significantly affects local haemodynamic forces. For the first time in this study, we performed pulsatile blood flow simulation taking into account the non-Newtonian nature of blood and implemented a meticulous methodology to assess the ESS and SR metrics at the top of and around the struts in the REFINED and conventional methodologies. The ESS values were higher in the REFINED model at the top of the apposed struts compared to the conventional approach in both *in vitro* and *in vivo* models, and the SR were more heterogeneous (as indicated by larger standard deviations in the SR values), indicating that the REFINED method can more accurately capture flow disturbances caused by protruding struts (Figure 2). Around malapposed struts, the ESS values were similar, but the SR values were higher and more heterogeneous with the REFINED approach compared to the conventional method, which was unable to reconstruct these struts. When the analysis focused on the multidirectional ESS, we found lower RRT on the top and higher RRT around the apposed struts with the REFINED method compared to the conventional approach. In malapposed struts, the RRT was higher on the lumen surface at the location of the projected strut with the REFINED method compared to the conventional approach, which was unable to incorporate these struts into the stented segment geometry or assess the implications of flow diversion and stagnation caused by the struts. This unfavourable milieu is likely to promote plaque progression in these segments, leading to strut coverage in the short term (38). Differences were also noted in the transESS values, which were higher at the top of the struts in apposed struts in the REFINED approach, and this should be attributed to the flow disturbances caused by the protruding struts. In malapposed struts, the transESS values were lower in the REFINED approach compared to the conventional approach. Conversely, there was no difference in the OSI values, which were close to zero in the areas of interest (Supplementary Figure 3). The disturbed flow patterns around the protruding and malapposed struts, captured by the REFINED method, can trigger the unfolding of the von Willebrand factor and platelet activation, hence corroborating the high risk of stent thrombosis reported in cases of large strut malapposition (39).

Finally, for the first time, we introduced a thrombus silicone model to evaluate the SR estimations of the two approaches in thrombosed and non-thrombosed volumes. We found a higher SR in the thrombus formed at the stenosis site in both reconstruction approaches. In the four cases where a thrombus was formed proximally to the stenosis, we found a larger proportion of the studied volume exposed to high and low SR in the REFINED approach compared to the conventional methods. The inability of the conventional method to incorporate the malapposed struts led to lower and more homogeneous SR in these regions, while in the REFINED approach, the malapposed struts generated flow disturbances, resulting in higher and more heterogeneous SR. These findings indicate that the REFINED approach is superior to the conventional method in detecting flow disturbances in cases of strut malapposition, which could potentially predict thrombus formation. This is because high and heterogeneous SR have been associated with platelet displacement towards the lumen surface, platelet activation, unfolding of the von Willebrand factor and platelet collision (18, 40, 41).

### Limitations

We acknowledge the following limitations of this study. First, the REFINED methodology, in the current form, cannot handle overlap and entangled struts in complicated bifurcation stenting such as Culotte or D-K crush techniques. Further developments are needed to reconstruct a stent deployed in a geometry significantly different from the original architecture. Second, the *in vitro* and *in vivo* assessments of the reconstructed stents were conducted using the 3D locations of the original strut points rather than an independently acquired reference standard (e.g., the deployed stent geometry captured by micro-computed tomography) (30, 33, 36). However, it has to be acknowledged that although micro-computed tomography can provide an undeniable reference standard for *in vitro* validation, it gives no information about the performance of the REFINED approach *in vivo*. For this purpose, we applied the same validation protocol *in vivo* and *in vitro*. Despite the cardiac motion and the forward-backwards motion of the OCT probe during the cardiac cycle, we found a small error distance between the stent model and the 3D location of the struts in the REFINED method, although this was larger than that reported *in vitro*. This finding gives us confidence about its ability to reconstruct stented segments *in vivo* (42). Third, we assessed the ESS and SR distributions using an arbitrary sampling area/volume around the struts (400 μm for the ESS and 320 μm for the SR), which were determined such that the heterogeneity of the biomechanical variables would be clearly depicted after a sensitivity analysis; an exploration towards its physiological justification is warranted. Fourth, coronary reconstruction was performed at one point in the cardiac cycle without considering the vessel motion that could affect the distribution of the local haemodynamic forces (43). In addition, we assumed that the vessel wall was rigid; this assumption is valid for embedded or apposed struts, but not for malapposed struts. Nevertheless, even in these locations, changes in lumen dimensions are expected to be minimal, as stents are deployed in diseased segments where the vessel wall distensibility is restricted (44). Moreover, although the *in vivo* simulations provided unique insights into the association between SR and thrombus formation, along with the efficacy of the REFINED method in detecting flow disturbances, it has to be acknowledged that our findings cannot be translated to *in vivo* settings for the following two reasons: 1) in the silicone models we performed steady flow rather than pulsatile flow simulations – to align with the experimental setup and 2) more importantly, the silicone models lack the natural thromboresistant properties of the living coronary arteries. Finally, both the REFINED and the conventional approaches are susceptible to artefacts induced by the cardiac motion and the motion of the OCT probe within the vessel during the cardiac cycle. However, their effect is expected to be minimal in more recent OCT systems with faster pull-back speeds, which are expected to enable assessment of the stented segments within a fraction of a cardiac cycle.

## Conclusions

We introduced and extensively evaluated a method for accurate reconstruction of stented segments that outperforms the conventional approach in representing stent geometry and assessing its implications on the local hemodynamic forces. This approach is expected to significantly enhance the accuracy of computed flow patterns in different stent designs, including emerging ones under development, and allow us to assess their implications on stent failure, such as neointimal proliferation and thrombus formation.

## Data Availability

The raw data supporting the findings of this study will be available from the corresponding authors upon reasonable request.
