# Supplementary material for: Realistic anatomical model reconstruction and high-fidelity flow computation in stented coronary arteries for the analysis of in-stent thrombosis
Source: Front Cardiovasc Med. 2026 Jul 23;13:1863952. doi: 10.3389/fcvm.2026.1863952 (PMC13442026; doi:10.3389/fcvm.2026.1863952)
Supplement: Supplementary file 1 [file Datasheet1.docx]

**Realistic anatomical model reconstruction and high-fidelity flow computation in stented coronary arteries for analysis of in-stent thrombosis**

Ryo Torii,^1,*^ PhD; Amear Mohamed,^1^ MEng; Mazen Abou Gamrah,^2^ PhD; Ibrahim Halil Tanboga,^3^ MD PhD; Zaid A. Abdulelah,^2^ MD; Anantharaman Ramasamy,^2,4^ MBChB, PhD; Thamil Kumaran,^2^ PhD; Tingquan Zhou,^2^ MD; Xingwei He,^2^ MD; Tom Crake,^2^ MD; Janna Leung,^5^ MBChB; Hui Ying Ang,^6,7^ PhD; Nicolas Foin,^6^ PhD; Jaryl Ng^6^, PhD; Shawn Lian Shaoliang,^6^ PhD; Pieter Kitslaar,^8^ MSc; Jouke Dijkstra,^9^ PhD; James Moon,^2,10^ MD; Andreas Baumbach,^2,4^ MD; Christos V. Bourantas,^2,4,**^ MD, PhD

^1^ Department of Mechanical Engineering, University College London, London, UK

^2^ Department of Cardiology, Barts Heart Centre, Barts Health NHS Trust, London, UK

^3^ Department of Biostatistics and Cardiology, Nisantasi University Medical School, Istanbul, Turkey

^4^ William Harvey Research Institute, Queen Mary University London, UK

^5^ Medical School, University of Birmingham, Birmingham, UK

^6^ National Heart Research Institute, Singapore, Singapore

^7^ Duke-NUS Medical School, Singapore, Singapore

^8^ Medis Medical Imaging Systems, Leiden, The Netherlands

^9^ Division of Image Processing, Department of Radiology, Leiden University Medical Center, Leiden, The Netherlands

^10^ Institute of Cardiovascular Sciences, University College London, London, UK

***Address for correspondence**

* Ryo Torii, PhD

Professor, Department of Mechanical Engineering, University College London

Torrington Place, London, UK, WC1E 7JE

E-mail: r.torii@ucl.ac.uk

Phone: +44 (0)20 7679 7062, Fax: +44 (0)20 7388 0180

** Christos Bourantas, MD, PhD

Consultant Cardiologist, Barts Heart Centre,

Barts Heart Centre, West Smithfield, London EC1A 7BE

E-mail: cbourantas@gmail.com

Phone: +44 20 7377 7000, Fax: +44 20 7791 9670

**Supplement on Method**

*Full in vitro study protocol*

Six stenotic vessel models were created for *in vitro* validation, with silicone elastomer (MED-4735 with 35 Shore hardness; NuSil Technology LLC, Carpinteria, CA, USA). All models had a reference diameter of 2.75 mm and a 40% diameter stenosis in their middle. The models had a 0.45 mm wall with isotropic elastic material properties (density 1110 kg/m^3^, Young’s modulus 1.2 MPa, Poisson’s ratio 0.48) and were implanted with a 3.0×18 mm sirolimus-eluting stent (Orsiro, Biotronik, Germany). In 3 models, the stents were deployed at the stenosis site with all the stent struts being well apposed, while in the remaining 3 models, the stents were deployed with either 1 mm or 5 mm length of stent struts left malapposed at the proximal segment of the stent before the stenotic site (malapposed distance ≈0.25 mm). In this way, 6 models were generated with 40% stent under-expansion with the presence of malapposition in 3 cases (1). The silicone models were perfused with porcine blood (containing 10% acid-citrate-dextrose anticoagulant) for 60 min at a flow rate of 200 ml/min; then the models were flushed with Tyrode’s solution to remove excess blood, and finally OCT imaging was performed using the C7XR (Abbott Vascular Inc, Santa Clara, CA, USA) system at a constant pullback speed of 20 mm/s to image the thrombosis generated in the models.

*Optical coherence tomography data processing*

The OCT data collected *in vitro* and *in vivo* were analysed offline using proprietary software (QCM-CMS Medis Medical Imaging System, Leiden, The Netherlands). For the *in vitro* silicone models, all the OCT frames portraying the model were segmented. In the OCT obtained *in vivo*, the stented segment was first identified, then the segment of interest was defined between the most proximal and distal side branches around the stented segment that were visible in both OCT and X-ray angiography. The OCT frames portraying the non-stented segment were analysed at 0.4 mm interval and the lumen borders were annotated. For the stented segment, analysis was performed at every 0.2 mm interval – as a previous study has shown that an increased longitudinal resolution (i.e., 0.1 mm) does not improve the accuracy of stent reconstruction (2) – and in each frame a unified lumen-stent border was annotated by following the lumen border where struts were embedded or malapposed and the top of the struts in case of apposed struts (Figure 1 C in the main text). Moreover a 2^nd^ analysis was performed in these frames that included identification of the stent struts and their annotation at the middle of the hyperintense signal, and detection of the lumen border that was defined by the innermost leading edge of the intima; when this was not visible, because of the shadowing artefacts behind the struts, the lumen was extrapolated from the closed identifiable lumen border (Figure 1 D in the main text) (3). Finally, thrombus was noted in some of the silicone models and in these cases the thrombus border was drawn when it was present.

*Reconstruction of coronary artery anatomical model*

In the conventional method the following steps were followed to reconstruct stented segment geometry:

1) Extraction of the lumen centreline. Two end-diastolic angiographic projections – ideally orthogonal but with an angle difference >25^o^ – acquired at the end of the procedure with limited foreshortening and no overlapping of the segment of interest were selected and processed by a well-validated 3D quantitative coronary angiography (3D-QCA) software (QAngioXA3DRE, Medis Medical Imaging Systems, Leiden, the Netherlands). In brief, after image calibration, the lumen borders of the segment of interest were automatically identified in the two projections using an established edge detection algorithm, these borders were used to define the lumen centreline and from these two centrelines a 3D centreline of the segment of interest was extracted. For silicone models, no angiographic images were acquired, and the centrelines were approximated by a straight line as the models were straight tubes.

2) Placement of the OCT lumen for the non-stented segment and of the unified lumen-stent borders for the stented segment perpendicularly onto the 3D vessel centreline.

3) Estimation of their relative twist using the sequential triangulation algorithm.

4) Extraction of the absolute orientation of the first OCT frame using anatomical landmarks (i.e. side branches) that were visible in both X-ray angiography and OCT imaging.

5) Fitting a 3D-surface on the point cloud generated from the above process that corresponded to the stented segment geometry.

6) In case of side branches originating from the segment of interest with a diameter >1mm 3D-QCA was performed to reconstruct their geometry, for a length of at least 25mm, and then the side branch models were connected and incorporated in the segment of interest.

In the REFINED method the lumen borders, instead of the unified lumen-stent borders annotated in stented segments, were merged with the angiographic data to reconstruct the lumen geometry of the stented segment and this was fused with he stent model generated by the stent struts to create the final lumen-stent 3D reconstruction.

*Evaluation of the performance of the two approaches in reconstructing stented segment geometry*

The performance of the two approaches to reconstruct stented segments was assessed *in vitro* and *in vivo* using the 3D co-ordinates of the original stent struts as a reference point. The reference points were obtained by translating the strut points originally found in OCT (i.e. strut centre point) images into 3D space, and the evaluation was conducted for every reference point. The strut reference points were first classified based on their relative location to the lumen border: as embedded if the strut was located outside the lumen border, malapposed if the strut was located inside the lumen border with its distance greater than the strut thickness (80mm in the Orsiro, 89µm in the Xience and 74µm in the Synergy stent) plus the radial resolution of OCT (14 µm) (4), and apposed otherwise; $D_{1,2,3}$ in Supplementary Figure 1. For the conventional approach, we measured the Euclidian distances between the reference points and the nearest points of the reconstructed stent and lumen geometry (also $D_{1,2,3}$ in Supplementary Figure 1 A). For the REFINED approach, the Euclidean distance between the reference points and the 3D stent geometry was measured ($d_{1,2,3}$ in Supplementary Figure 1 B). These distances were referred as error distances.

*Comparison of the flow patterns in the models reconstructed by the two methodologies*

The impact of the two reconstruction approaches on flow patterns was evaluated by measuring the ESS and SR: (i) on the struts (sampling area/volume = strut thickness) and (ii) around the struts (sampling area/volume = 400 μm) – illustrated in Figure 3 (main text).

For each strut point, the closest point from the lumen surface in the conventional approach was identified and this was used to define two sampling squares on the lumen surface with a side equal to the strut dimensions and 400 μm respectively; the first corresponded to the top of the strut and the second to the peri-strut area where flow disturbances are expected (Figure 3 in the main text). Similarly in the REFINED approach, we identified for each strut point the closest point to the stent model when this was apposed, while in case of strut embedment or malapposition, we detected its closed point on the lumen surface and this was used to define two sampling squares with a side equal to the strut dimensions and 400 μm, respectively. In the *in vitro* models, the ESS was measured in these areas. In the *in vivo* models, where pulsatile flow was imposed, the time averaged ESS (TAESS) and the following multi-directional ESS metrics were calculated: oscillatory shear index (OSI), relative residence time (RRT), transverse ESS (transESS) and cross flow index (CFI) (5). Additionally, each stent strut point was used to define a sphere with a diameter of 320 μm and in this volume we measured the mean SR and its spatial standard deviation (Figure 3 in the main text). Finally, in the silicone models, we measured the SR in the thrombus volume and the non-thrombosed segment in the models reconstructed by the two methodologies.

**Supplementary Table 1.** Baseline demographics of the studied patients.

| **Studied patients** | **N=16** |
| --- | --- |
| Age | 57.9 ± 13.4 |
| Gender (Male) | 13 (81.3%) |
| Current or ex smoker | 10 (62.5%) |
| **Co-morbidities** |  |
| Diabetes mellitus | 6 (37.5%) |
| Hypertension | 9 (56.25%) |
| Hypercholesterolemia | 10 (62.5%) |
| Renal failure* | 0 |
| Previous PCI | 7 (43.8%) |
| LV function |  |
| Good LV function | 11 (68.8%) |
| Impaired LV function** | 5 (31.3%) |
| **Total number of treated vessels** | **N=18** |
| LAD/D1*** | 12 (66.7%) |
| LCx | 4(22.2%) |
| RCA | 2 (11.1%) |
| **Deployed stents** | **N=21** |
| Synergy | 2 (9.5%) |
| Xience | 19(90.5%) |
| **Medications at discharge** | |
| β-blockers 13 (81.25%) | |
| RAAS inhibitors 12(75%) | |
| Statins 12(75%) | |
| Aspirin | 15 (94.7%) |
| Ticagrelor | 6 (37.5%) |
| Clopidogrel | 9 (52.3%) |

**Table footnote:** D1, diagonal branch; LAD, left anterior descending artery; LCx, left circumflex artery; LV, left ventricle; PCI, percutaneous coronary intervention; RAAS, renin angiotensin aldosterone system inhibitors; RCA, right coronary artery.

*Renal failure is defined as an estimated glomerular filtration rate of <60ml/min/1.73m2

**Impaired LV function was defined as LV ejection fraction of <50%

***In two cases disease involved the diagonal and this PCI was performed in both vessels with T and small protrusion technique.

**Supplementary Table 2.** Distributions of the ESS-based metrics based on the two reconstruction methods in the in vivo data.

|  | **Conventional** | **REFINED** | **H-L estimate (95% CI)** | **p-value** |
| --- | --- | --- | --- | --- |
| **RRT on the top of struts** | |  |  |  |
| Entire set of struts | 0.93 (0.53, 1.86) | 0.56 (0.34, 0.98) | 0.51 (0.49, 0.52) | <0.001 |
| Apposed struts | 0.91 (0.50, 1.89) | 0.47 (0.31, 0.77) | 0.56 (0.54, 0.58) | <0.001 |
| Embedded struts | 0.92 (0.61, 1.54) | 0.89 (0.59, 1.39) | 0.20 (0.16, 0.24) | <0.001 |
| Malapposed struts | 1.32 (0.60, 2.95) | 1.47 (0.57, 3.48) | -0.40 (-0.68, -0.19) | <0.001 |
| **RRT around struts** |  |  |  |  |
| Entire set of struts | 1.27 (0.77, 2.36) | 1.38 (0.82, 2.63) | -0.01 (-0.02, -0.01) | <0.001 |
| Apposed struts | 1.41 (0.83, 2.59) | 1.59 (0.92, 2.93) | -0.05 (-0.05, -0.04) | <0.001 |
| Embedded struts | 0.93 (0.64, 1.50) | 0.91 (0.63, 1.38) | 4x10^-4^ (2x10^-4^, 5x10^-4^) | <0.001 |
| Malapposed struts | 1.37 (0.75, 2.92) | 1.59 (0.70, 3.17) | -5x10^-4^ (-8x10^-4^, -3x10^-4^) | <0.001 |
| **OSI on the top of struts** | | | | |
| Entire set of struts | 3x10^-4^ (5x10^-5^, 2x10^-2^) | 2x10^-4^ (4x10^-5^, 1x10^-3^) | 1x10^-5^ (2x10^-5^, 5x10^-5^) | <0.001 |
| Apposed struts | 2x10^-4^ (5x10^-5^, 2x10^-3^) | 2x10^-4^ (4x10^-5^, 9x10^-4^) | 7x10^-5^ (6x10^-5^, 8x10^-5^) | <0.001 |
| Embedded struts | 4x10^-4^ (6x10^-5^, 2x10^-3^) | 3x10^-4^ (6x10^-5^, 1x10^-3^) | 3x10^-4^ (2x10^-4^, 4x10^-4^) | <0.001 |
| Malapposed struts | 2x10^-3^ (3x10^-4^, 7x10^-3^) | 2x10^-3^ (2x10^-4^, 7x10^-3^) | -4x10^-5^ (-4x10^-4^, 1x10^-4^) | 0.531 |
| **OSI around struts** |  |  |  |  |
| Entire set of struts | 6x10^-4^ (1x10^-4^, 3x10^-3^) | 6x10^-4^ (1x10^-4^, 3x10^-3^) | 1x10^-4^ (4x10^-5^, 6x10^-5^) | <0.001 |
| Apposed struts | 6x10^-4^ (1x10^-4^, 3x10^-3^) | 6x10^-4^ (1x10^-4^, 3x10^-3^) | 1x10^-6^ (-2x10^-5^, 5x10^-5^) | 0.002 |
| Embedded struts | 4x10^-4^ (9x10^-5^, 2x10^-3^) | 4x10^-4^ (7x10^-5^, 2x10^-3^) | 6x10^-5^ (2x10^-5^, 7x10^-5^) | <0.001 |
| Malapposed struts | 3x10^-3^ (6x10^-4^, 8x10^-3^) | 3x10^-3^ (7x10^-4^, 9x10^-3^) | -2x10^-5^ (-3x10^-5^, -1x10^-5^) | <0.001 |
| **TransESS on the top of strut** | |  |  |  |
| Entire set of struts (Pa) | 0.03 (0.01, 0.06) | 0.04 (0.02, 0.09) | -0.013 (-0.013, -0.012) | <0.001 |
| Apposed struts (Pa) | 0.03 (0.01, 0.06) | 0.05 (0.02, 0.10) | -0.015 (-0.016, -0.015) | <0.001 |
| Embedded struts (Pa) | 0.03 (0.02, 0.06) | 0.03 (0.02, 0.06) | 0.002 (0.001, 0.003) | <0.001 |
| Malapposed struts (Pa) | 0.04 (0.01, 0.08) | 0.03 (0.01, 0.07) | 0.022 (0.015, 0.029) | <0.001 |
| **TransESS around strut** | |  |  |  |
| Entire set of struts (Pa) | 0.03 (0.02, 0.06) | 0.03 (0.01, 0.05) | -2x10^-3^(-3x10^-3^, -2x10^-3^) | <0.001 |
| Apposed struts (Pa) | 0.03 (0.01, 0.06) | 0.03 (0.01, 0.05) | 6x10^-4^(6x10^-4^, 7x10^-4^) | <0.001 |
| Embedded struts (Pa) | 0.03 (0.02, 0.06) | 0.03 (0.02, 0.06) | 4x10^-5^(2x10^-5^, 5x10^-5^) | 0.045 |
| Malapposed struts (Pa) | 0.04 (0.02, 0.09) | 0.04 (0.02, 0.08) | 3x10^-5^(-1x10^-6^, 2x10^-5^) | 0.126 |
| **CFI on the top of struts** | |  |  |  |
| Entire set of struts | 0.03 (0.01, 0.06) | 0.02 (0.01, 0.05) | 0.003 (0.002, 0.003) | <0.001 |
| Apposed struts | 0.02 (0.01, 0.06) | 0.02 (0.01, 0.05) | 0.002 (0.002, 0.002) | <0.001 |
| Embedded struts | 0.03 (0.01, 0.07) | 0.02 (0.01, 0.06) | 0.010 (0.008, 0.012) | <0.001 |
| Malapposed struts | 0.05 (0.02, 0.11) | 0.05 (0.02, 0.10) | 0.010 (0.004, 0.016) | <0.001 |
| **CFI around struts** | |  |  |  |
| Entire set of struts | 0.03 (0.02, 0.07) | 0.03 (0.01, 0.06) | 3x10^-4^(1x10^-4^, 3x10^-4^) | <0.001 |
| Apposed struts | 0.03 (0.01, 0.06) | 0.03 (0.01, 0.06) | 4x10^-4^(3x10^-4^, 5x10^-4^) | <0.001 |
| Embedded struts | 0.03 (0.02, 0.07) | 0.03 (0.01, 0.06) | 5x10^-5^(5x10^-5^, 5x10^-5^) | <0.001 |
| Malapposed struts | 0.05 (0.03, 0.10) | 0.05 (0.03, 0.09) | -2x10^-5^(1x10^-5^, 2x10^-5^) | 0.231 |

**Table footnote:** CFI, cross flow index; OSI, oscillatory index; RRT, relative residence time; TAESS, time average endothelial shear stress; TransESS, transverse endothelial shear stress.

**Supplementary Figure 1.** Methodology implemented to test the performance of the reconstruction approaches in generating geometrically correct 3D models. In the conventional approach (Panel A), the Euclidean distance between the 3D location of the struts and the lumen-stent model is computed in embedded (D_1_), malapposed (D_2_) and apposed struts (D_3_) and these also represent the error in model reconstruction. In the REFEND approach (Panel B) the distance between the 3D location of the struts and the stent model is computed (d_1_, d_2_, d_3_) to estimate the error in model reconstruction and all strut apposition categories that are determined by taking into account the strut thickness and the distance (D) between the 3D location of the strut and the lumen surface.

**Supplementary Figure 2.** Euclidean distance between the 3D location of the struts and the lumen-stent model in the conventional approach for the entire set of struts (A) and for apposed (B) embedded (C) and malapposed struts (D) and between the stent model and the 3D location of the strut in the REFINED approach for the entire strut sent and for the three strut apposition categories.

**Supplementary Figure 3.** Spread-out plots showing the estimations of the conventional and the REFINED approach for the TAESS (A, B), RRT (C, D), OSI (E, F), transESS (G, H) and CFI (I, J) in a stented segment

**References**

1.	Ng JCK, Lian SS, Zhong L, Collet C, Foin N, Ang HY. Stent Malapposition Generates Stent Thrombosis: Insights from a Thrombosis Model. *Int J Cardiol* (2022) 353:43–5. Epub 20220208. doi: 10.1016/j.ijcard.2022.02.003.

2.	O'Brien CC, Kolandaivelu K, Brown J, Lopes AC, Kunio M, Kolachalama VB, et al. Constraining Oct with Knowledge of Device Design Enables High Accuracy Hemodynamic Assessment of Endovascular Implants. *PLoS One* (2016) 11(2):e0149178. Epub 2016/02/26. doi: 10.1371/journal.pone.0149178.

3.	Nakatani S, Sotomi Y, Ishibashi Y, Grundeken MJ, Tateishi H, Tenekecioglu E, et al. Comparative Analysis Method of Permanent Metallic Stents (Xience) and Bioresorbable Poly-L-Lactic (Plla) Scaffolds (Absorb) on Optical Coherence Tomography at Baseline and Follow-Up. *EuroIntervention* (2016) 12(12):1498–509. Epub 2015/10/09. doi: 10.4244/EIJY15M10_03.

4.	Gutierrez-Chico JL, Regar E, Nuesch E, Okamura T, Wykrzykowska J, di Mario C, et al. Delayed Coverage in Malapposed and Side-Branch Struts with Respect to Well-Apposed Struts in Drug-Eluting Stents: In Vivo Assessment with Optical Coherence Tomography. *Circulation* (2011) 124(5):612–23. Epub 2011/07/20. doi: 10.1161/CIRCULATIONAHA.110.014514.

5.	Kok AM, Molony DS, Timmins LH, Ko YA, Boersma E, Eshtehardi P, et al. The Influence of Multidirectional Shear Stress on Plaque Progression and Composition Changes in Human Coronary Arteries. *EuroIntervention* (2019) 15(8):692–9. doi: 10.4244/EIJ-D-18-00529.
